# Graphene Oxide in Lossy Mode Resonance-Based Optical Fiber Sensors for Ethanol Detection

**DOI:** 10.3390/s18010058

**Published:** 2017-12-27

**Authors:** Miguel Hernaez, Andrew G. Mayes, Sonia Melendi-Espina

**Affiliations:** 1 School of Chemistry, University of East Anglia, Norwich Research Park, Norwich NR4 7TJ, UK; andrew.mayes@uea.ac.uk; 2 Engineering Division, School of Mathematics, University of East Anglia, Norwich Research Park, Norwich NR4 7TJ, UK

**Keywords:** optical fiber sensor, ethanol sensor, lossy mode resonances, graphene oxide, layer-by-layer

## Abstract

The influence of graphene oxide (GO) over the features of an optical fiber ethanol sensor based on lossy mode resonances (LMR) has been studied in this work. Four different sensors were built with this aim, each comprising a multimode optical fiber core fragment coated with a SnO_2_ thin film. Layer by layer (LbL) coatings made of 1, 2 and 4 bilayers of polyethyleneimine (PEI) and graphene oxide were deposited onto three of these devices and their behavior as aqueous ethanol sensors was characterized and compared with the sensor without GO. The sensors with GO showed much better performance with a maximum sensitivity enhancement of 176% with respect to the sensor without GO. To our knowledge, this is the first time that GO has been used to make an optical fiber sensor based on LMR.

## 1. Introduction

Graphene has become a trending topic in different scientific fields since Novoselov and Geim reported its successful isolation in 2004 [1]. Its outstanding properties make it an ideal candidate for many applications. However, the complexity and price of its synthesis make the use of graphene unrealistic in some fields. In this regard, graphene oxide (GO) and reduced graphene oxide (rGO) can be good substitutes of graphene in many applications sharing many of the interesting properties of graphene but with easier production, access to a wide variety of deposition methods and more affordable cost. In particular, GO and rGO are attracting increasing attention in recent years for sensing applications due to their high specific surface area, high electronic mobility and low electrical noise [2,3,4,5].

Among all sensing strategies, optical fiber sensors have achieved a high impact in the last decades because they offer several advantages over electronic sensors [6,7,8,9]. Particularly, optical fiber sensors are light, small, resistant to harsh environments and high temperatures, biocompatible, immune to electromagnetic fields and electromagnetically passive. These features make them especially suitable for some specific applications, such as biosensing, health care and sensing in offshore, harsh or flammable environments.

In this respect, resonance-based sensing devices are attracting the interest of the scientific community due to their versatility, high sensitivity and robustness [10,11]. In particular, lossy mode resonance (LMR) constitutes a promising research field that could compete with the largely established surface plasmon resonance-based sensors, since they do not depend on the polarization of light and enlarge the range of materials available for sensor surface coating from noble metals to metal oxides, polymers and many others [12,13,14]. When an optical waveguide is coated with a thin-film, the propagation of light is affected. If the refractive index of the coating has a non-zero imaginary part, it introduces losses that can produce electromagnetic resonances. Depending on the properties of the waveguide, the coating and the external medium, different cases of electromagnetic resonances can be distinguished [15,16], LMR among them. These LMRs produce an absorption band in the transmitted spectrum at a determined wavelength. A shift of this resonance peak can be observed when the optical conditions of the system change, i.e., when there is a variation in the refractive index of the coating or the surrounding medium. Consequently, if a thin-film sensitive to some analyte is deposited onto a waveguide and used as resonance-supporting coating, a variation in the concentration of this analyte will produce a measurable shift of the LMR peak [12,17,18,19,20].

Ethanol is commonly utilized in many fields, such as medicine, food processing, beverage industry or even as an increasingly important bio-fuel [21,22]. Hence, detection of ethanol concentration in water is an interesting field with particular importance for the mentioned sectors. Most available commercial ethanol sensors are electrical transducers based on resistance and voltage [23,24]. However, the high flammability and volatility of ethanol make optical fiber sensors an ideal candidate for safe and accurate measurement of this substance [25,26,27,28,29,30,31].

The aim of this work was to confirm the suitability of GO coatings to improve the performance of LMR-based optical fiber sensors. To reach this goal, LMR optical fiber ethanol sensors based on a sputtered SnO_2_ coating were fabricated and characterized. These were modified by depositing a multilayer coating including polyethyleneimine (PEI) and GO onto the SnO_2_ layer following the layer-by-layer (LbL) technique [32,33,34,35]. Finally, these devices were characterized and their responses compared in terms of sensitivity, linearity and hysteresis. 

## 2. Materials and Methods 

The experimental aspects of this research, such as the fabrication of the sensing devices and the characterization setup and procedure are explained in this section.

### 2.1. Sensor Fabrication

The fabrication of the sensors involves several steps. Firstly, a 10 cm fragment of a 200 µm-core multimode optical fiber (FT200EMT, purchased from Thorlabs, Inc., Ely, UK) was prepared. Its cladding was thermally removed and the resultant bare core was sonicated and cleaned in both detergent and acetone and rinsed with ultrapure water. After the cleaning process, it was coated during 150 s with SnO_2_ using a sputter coater (K675XD from Quantum Technologies) [36]. In order to obtain an even film, the substrate was subjected to continuous rotation during the sputtering process. Four 2 cm fragments of the resultant device were perpendicularly cleaved using a NorthLab ProCleave LD II. Each of them was spliced to 200 μm optical fiber core pigtails in both extremes using a Fitel S178A fusion splicer in order to connect it to the rest of the characterization setup. One of these devices was reserved and called S0 from this point.

Sensors SGO1b, SGO2b and SGO4b are the result of depositing a thin film consisting of one, two and four bilayers respectively of PEI/GO onto the remaining SnO_2_-coated optical fiber fragments. 

The layer-by-layer deposition method was applied to deposit these coatings. A 0.5 mg mL^−1^ GO (Graphenea, S.A., San Sebastian, Spain) solution and 2 mg mL^−1^ PEI (Sigma-Aldrich) solution in DI water were prepared and left stirring overnight. Just before starting the deposition, the GO solution was sonicated for 2 h to avoid aggregation. At the same time, the sputtered fiber was immersed into a 1M-KOH solution for 30 min, then thoroughly rinsed with DI water.

The LbL deposition started with the immersion of the substrate into the PEI solution for 5 min. After this time, the substrate was rinsed with DI water for 3 min in order to remove the excess of material and dried in air for 3 min. Then, the substrate was immersed into the GO solution to deposit the first bilayer of PEI/GO. The final coating consisted of the repetition of the whole LbL process until reaching the desired number of bilayers.

### 2.2. Sensors Characterization

A FEI NanoSEM 450 FEG SEM scanning electron microscope was used to obtain an image of the cross section of sensor S0.

1, 2 and 4-bilayer coatings of PEI/GO were also deposited onto glass slides, following the same deposition procedure, with the aim of studying their thickness, morphology and texture. A Veeco Innova atomic force microscope (AFM) and the FEI SEM mentioned above were used to obtain images of these coatings. In order to measure the thicknesses of the coatings, a narrow scratch (~20 μm wide) was made on the surface of each film using a scalpel. An AFM analysis was performed covering the scratch and the adjacent areas on both sides. The thickness of the coating was measured as the difference in height between the substrate (scratched area) and the film (area adjacent to the scratch). This procedure was repeated in 4 different areas of each sample and the thickness of the coating was calculated as the average value of the 4 measurements.

**Figure 1:**
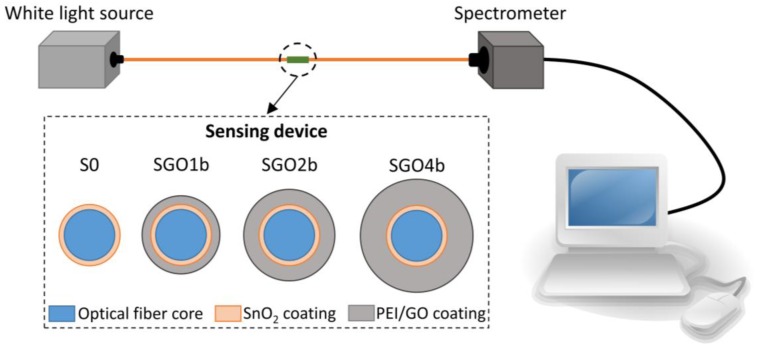
Optical fiber experimental setup and sensors schematic structure.

The typical optical fiber transmission setup shown in Figure 1 was used to collect the spectra of the light transmitted through the sensor. A halogen white light source (HL2000, Oceanoptics Inc., Oxford, UK) was connected to one of the optical fiber pigtails in order to couple light into the sensing device. The coupled light passes through the sensor and reaches a spectrometer (Oceanoptics USB2000) connected to the other pigtail. In order to characterize the response of the different devices as ethanol sensors, the sensitive fragments (S0, SGO1b, SGO2b and SGO4b) were immersed into aqueous ethanol solutions at *v/v* concentrations of 0%, 20%, 40%, 60%, 80% and 100% and the transmitted spectra were collected. In order to consistently calculate the wavelengths at which the LMR peaks are centered, a Matlab routine was designed. It returns the coefficients for a polynomial of degree 2 that is the best fit for each LMR peak. The wavelength at which the maximum of the curve is located is then numerically calculated.

Additionally, as sensor SGO4b showed the best sensitivity, it was selected to be dynamically characterized using the same experimental setup. With this aim, its sensitive part was sequentially immersed and withdrawn into a 40% *v/v* ethanol in water solution every 30 s. The absorption spectra were collected every 250 ms. A MatLab routine was created to obtain the LMR wavelength of each of these spectra.

## 3. Results

In this section, the characterization of the devices as ethanol sensors as well as the characterization of the PEI/GO coatings deposited to build sensors SGO1b, SGO2b and SGO4b are presented.

### 3.1. Deposition and Characterisation of the PEI/GO Coatings

As previously stated, three coatings consisting of 1, 2 and 4 bilayers of PEI/GO were deposited onto three SnO_2_-coated structures identical to S0 to obtain three additional sensors (SGO1b, SGO2b and SGO4b). 

**Figure 2:**
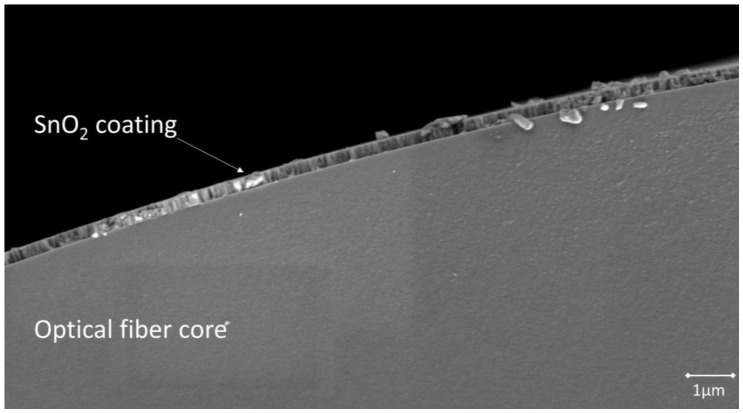
SEM image of S0 cross section. A homogeneous SnO_2_ coating can be clearly distinguished from the optical fiber core.

In Figure 2, a SEM image of the sensor S0 cross section is shown. This sensor was perpendicularly cleaved after being characterized to obtain this image. The SnO_2_ coating sputtered onto the optical fiber core presents a smooth and homogeneous aspect. It is clearly visible and shows an average thickness of 220 nm, measured in four different areas of the optical fiber circumference. 

**Figure 3:**
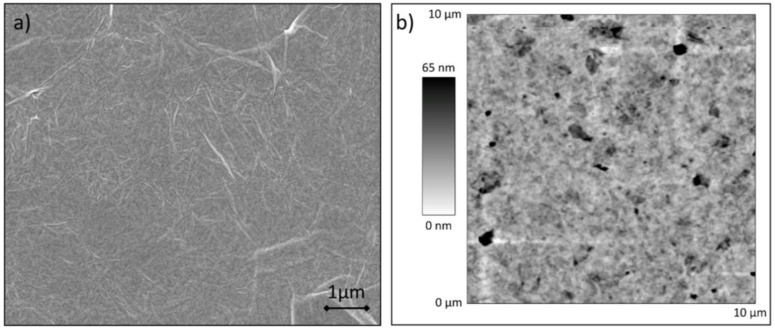
Images of the superficial structure of a 1-bilayer PEI/GO coating deposited onto glass slide: (**a**) SEM and (**b**) AFM.

In order to show the uniformity and quality of the deposited thin films onto the fiber core and to estimate film thickness more precisely, 1, 2 and 4-bilayer coatings of PEI/GO were deposited onto glass slides following the same procedure that was applied to deposit the sensitive coatings onto the optical fibers. The morphology of these films was characterized by means of FESEM and AFM. The homogeneous and uniform surface of the 1-bilayer PEI/GO coating is shown in Figure 3. Additionally, the characteristic folds of GO nanosheets can be clearly observed in these images. AFM analysis revealed an average thickness of 8, 17 and 31 nm for the coatings with 1, 2 and 4 bilayers, respectively.

### 3.2. Construction of the Sensors

**Figure 4:**
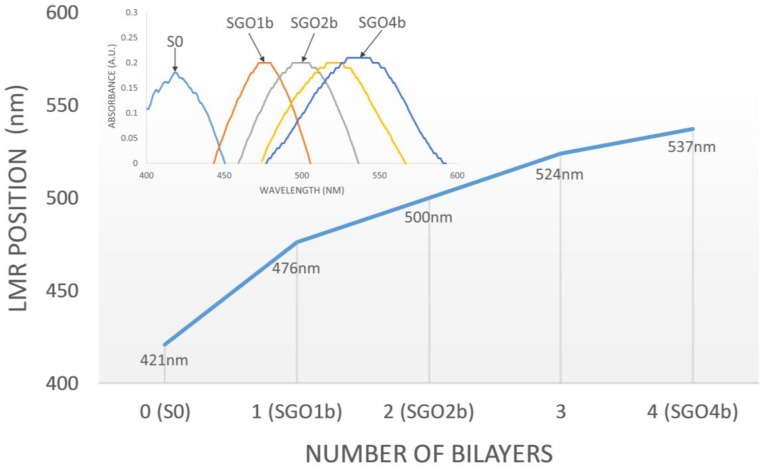
Shift of the LMR absorption peak generated by S0 during the deposition of four bilayers of PEI/GO.

In this part, the generation and the shift of the LMR absorption peak during the construction of the PEI/GO coatings onto the fiber core are presented. When S0 is connected to the experimental setup introduced in Figure 1, part of the optical power is transmitted through the coating and lost, generating a LMR [12,17,18,19,20]. Consequently, an absorption peak produced by this LMR can be observed in the transmitted spectrum captured at the spectrometer. As shown in Figure 4, when the sensitive device is in air, this absorption peak is centered at 421 nm.

The LMR generated by the SnO_2_ coating is highly sensitive to changes in the external refractive index, as was extensively studied and reported in [36]. For this reason, when a coating with refractive index higher than air is deposited onto it, it shifts to higher wavelengths. During the construction of the PEI/GO coating, the LMR peak with the sensing section in air shifts to 476 nm after the deposition of 1 bilayer, to 500 nm after 2 bilayers, to 524 nm after 3 bilayers and to 537 nm after 4 bilayers. This shift data is presented in Figure 4.

### 3.3. Response to Ethanol

The four fabricated optical fiber devices were characterized as ethanol sensors by introducing them into solutions of ethanol in water with concentrations from 0% (distilled water) to 100% (pure ethanol). The LMR absorption peaks generated at the SnO_2_ coating, and relocated when the PEI/GO thin films were deposited, shift to higher wavelengths when the refractive index of the external medium is increased. In this case, the increasing refractive index of the ethanol solutions will produce a shift to the right. The presence of the PEI/GO coatings enhances the sensitivity of the system because of the variation of their own effective refractive index as a function of the amount of ethanol absorbed.

**Figure 5:**
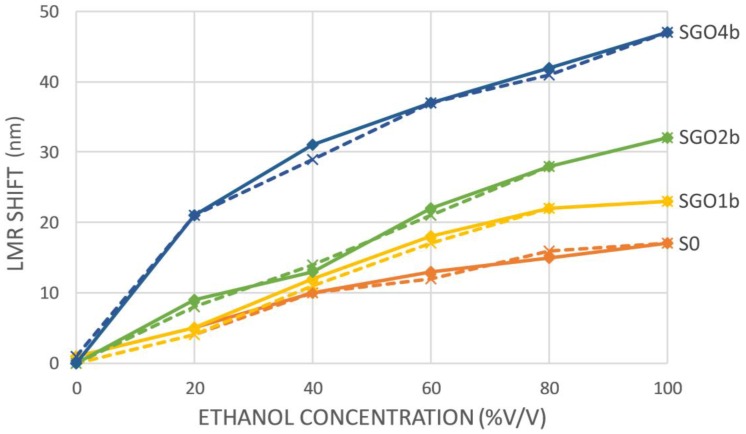
Shift of the LMR absorption peak generated by the different sensors when they are immersed in aqueous ethanol solutions with different concentrations from 0 to 100% *v/v* (straight lines) and from 100 to 0% *v/v* (dashed lines).

In Figure 5, the shifts in nm of S0, SGO1b, SGO2b and SGO4b LMR peaks are represented as a function of the ethanol concentration of the solutions. The responses recorded as ethanol concentrations were increased and then decreased again confirmed that the sensors present high repeatability and reversibility, improving other works found in the literature [27]. There was no obvious hysteresis in the response since the maximum variation between the measurements was only 2 nm and the distribution of the variance was random.

It is apparent that the LMR wavelength shift of the devices in the studied ethanol concentration range increases with the number of deposited PEI/GO bilayers. In particular, its value is 17 nm for the sensor without GO (S0, orange lines), 23 nm for the sensor with 1 bilayer of PEI/GO (SGO1b, yellow lines), 32 nm for the sensor with 2 bilayers (SGO2b, green lines) and 47 for the sensor with 4 bilayers (SGO4b, blue lines). In other words, these data corresponds to a shift increment of 35.29%, 88.24% and 176.47% in the studied range respectively, which leads to an improvement of sensitivity. This effect is even more important if we consider the ethanol concentration range from 0 to 40%. In this case, the shift is 10, 12, 14 and 31 nm with an enhancement of 20, 30 and 210% for SGOb, SGO2b and SGO4b, respectively. This range can be of particular interest for measuring ethanol concentration in alcoholic beverages or in fermentation processes for biofuel production. A summary of these results can be found in Table 1.

### 3.4. Dynamic Response

Once the static response of the four sensors was characterized and the better performance of SGO4b was confirmed, the dynamic response of this device was tested by introducing it into a 40% *v/v* ethanol water solution and withdrawing it to air every 30 s during 4 cycles.

**Figure 6:**
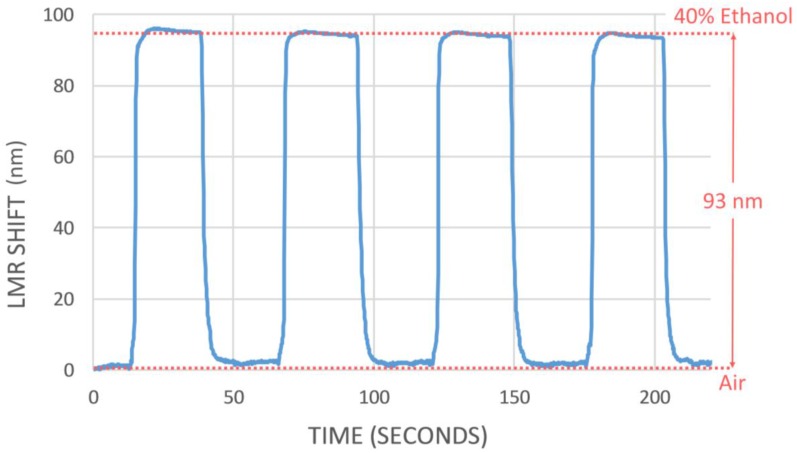
Dynamic response of SGO4b when it is immersed and withdrawn in a 40% *v/v* ethanol solution in water.

Figure 6 shows the results of this experiment. As it can be seen, the shift of the LMR peak when the external medium change from air to 40% ethanol is 93 nm and the response of the sensor is very fast and highly repetitive. It is worthy to note that the shift value shown in Figure 5 (30 nm for 40% ethanol) was obtained using as a reference the position of the LMR peak when the sensor is immersed into water. However, the shift presented in Figure 6 (93 nm) was measured between air and a 40% ethanol solution.

**Figure 7:**
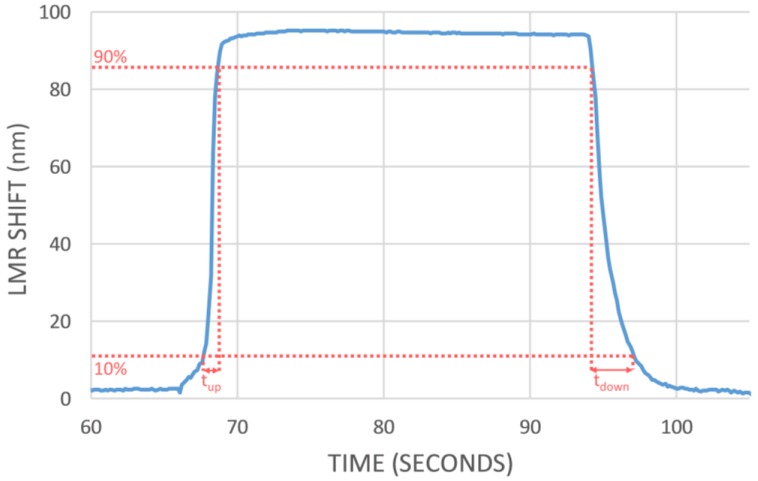
Single cycle of the dynamic response where the calculation of the rise time (t_up_) and the fall time (t_down_) is shown.

The second cycle, shown in Figure 6, has been amplified in Figure 7 in order to characterize the response time of the sensor. With this aim, the times between 10% and 90% of the total shift (t_up_) and between 90% and 10% (t_down_) have been calculated obtaining values of 1 s and 2.750 s respectively. To our knowledge, this is the first time that an optical fiber sensor based on LMR for ethanol concentration in water is reported so it is difficult to establish a comparison for these values. However, as a reference, recent works based on other phenomena showed rise and fall times of 40 and 70 s [25], 13 and 6 s [26], more than 10 min [37]. Authors could just find a work presenting similar dynamic times [27]. From these results, it can be concluded that the sensor presented here shows a fast reaction and recovery.

## 4. Conclusions

A significant increase in the sensitivity of an optical fiber ethanol sensor based on LMR has been achieved by depositing a multilayer coating made of PEI and GO onto the initial device. Four sensors based on a multimode optical fiber with the cladding removed, sputtered with a single layer of SnO_2_ were prepared. Three coatings consisting of 1, 2 and 4 bilayers of PEI/GO were deposited onto these initial sensors to obtain sensors SGO1b, SGO2b and SGO4b, respectively. S0 does not include any additional coating. These four devices were characterized by introducing them into aqueous ethanol solutions with concentrations of 0%, 20%, 40%, 60%, 80% and 100% *v/v*. S0 showed a dynamic range in the tested range of 17 nm. This value was found higher for SGO1b (23 nm), SGO2b (32 nm) and SGO4b (47 nm). In other words, wavelength shift of the LMR was increased in a 35%, an 88% and a 176% by just depositing 1, 2 and 4 bilayers of PEI/GO. The enhancement is even higher (20, 40 and 210%, respectively) if the range of ethanol concentrations is limited to 0 to 40%.

Additionally, the dynamic response of the sensor with the best sensitivity SGO4b was tested introducing and withdrawing it into a 40% ethanol solution in water. It shows a fast, repetitive response with a rise time of 1 s and a recovery time of 2.750 s. These values clearly improve on the results obtained in other recently reported studies using different sensor configurations.

To our knowledge, this is the first time that GO is included in an LMR-based optical fiber sensor, showing a very promising performance and potential.

## Figures and Tables

**Table 1:** Dynamic range of the four studied sensors.

Range	0 to 100% *v/v* Ethanol		0 to 40% *v/v* Ethanol	
Sensor	Shift (nm)	Enhacement	Shift (nm)	Enhacement
S0	17	-	10	-
SGO1b	23	35.29%	12	20%
SGO2b	32	88.24%	14	40%
SGO4b	47	176.47%	31	210%
